# Comparative genomics of *Campylobacter jejuni* from clinical campylobacteriosis stool specimens

**DOI:** 10.1186/s13099-022-00520-1

**Published:** 2022-12-07

**Authors:** Bilal Djeghout, Samuel J. Bloomfield, Steven Rudder, Ngozi Elumogo, Alison E. Mather, John Wain, Nicol Janecko

**Affiliations:** 1 Quadram Institute Bioscience, Rosalind Franklin Rd, Norwich Research Park, Norwich, NR4 7UQ UK; 2 Eastern Pathology Alliance, Norfolk and Norwich University Hospital, Norwich, NR4 7UY UK; 3 Faculty of Medicine and Health Sciences, University of East Anglia, Norwich, NR4 7TJ UK

**Keywords:** *Campylobacter jejuni*, Genomic diversity, Campylobacteriosis, Pangenomics

## Abstract

**Background:**

*Campylobacter jejuni* is a pervasive pathogen of major public health concern with a complex ecology requiring accurate and informative approaches to define pathogen diversity during outbreak investigations. Source attribution analysis may be confounded if the genetic diversity of a *C. jejuni* population is not adequately captured in a single specimen. The aim of this study was to determine the genomic diversity of *C. jejuni* within individual stool specimens from four campylobacteriosis patients. Direct plating and pre-culture filtration of one stool specimen per patient was used to culture multiple isolates per stool specimen. Whole genome sequencing and pangenome level analysis were used to investigate genomic diversity of *C. jejuni* within a patient.

**Results:**

A total 92 *C. jejuni* isolates were recovered from four patients presenting with gastroenteritis. The number of isolates ranged from 13 to 30 per patient stool. Three patients yielded a single *C. jejuni* multilocus sequence type: ST-21 (n = 26, patient 4), ST-61 (n = 30, patient 1) and ST-2066 (n = 23, patient 2). Patient 3 was infected with two different sequence types [ST-51 (n = 12) and ST-354 (n = 1)]. Isolates belonging to the same sequence type from the same patient specimen shared 12–43 core non-recombinant SNPs and 0–20 frameshifts with each other, and the pangenomes of each sequence type consisted of 1406–1491 core genes and 231–264 accessory genes. However, neither the mutation nor the accessory genes were connected to a specific functional gene category.

**Conclusions:**

Our findings show that the *C. jejuni* population recovered from an individual patient’s stool are genetically diverse even within the same ST and may have shared common ancestors before specimens were obtained. The population is unlikely to have evolved from a single isolate at the time point of initial patient infection, leading us to conclude that patients were likely infected with a heterogeneous *C. jejuni* population. The diversity of the *C. jejuni* population found within individual stool specimens can inform future methodological approaches to attribution and outbreak investigations.

**Supplementary Information:**

The online version contains supplementary material available at 10.1186/s13099-022-00520-1.

## Background

*Campylobacter* is one of the most common causes of bacterial gastroenteritis worldwide, outnumbering *Salmonella*, toxigenic *Escherichia coli* and *Listeria* combined [1, 2]. In the United Kingdom (UK), it is estimated that more than 299,000 cases occur annually [3, 4]. In most cases, infections are self-limiting, however, some cases result in persistent or invasive infections where antimicrobial therapy may be necessary [5–7]. Diagnosis is usually made by identification using PCR-based rapid detection assays or culture-based isolation of a single presumptive *Campylobacter* colony from a stool specimen [8], which does not identify co-infection with different *Campylobacter* species nor the presence of multiple sequence types [9]. With an estimated minimum infective dose of between 500 and 10,000 organisms, *C. jejuni* is responsible for 90% of known human campylobacteriosis infections [10–12]. Intraspecific recombination within *C. jejuni* is frequent [13]. Due to this, genetic exchange events are frequently overestimated in SNP analyses that compare strains at the nucleotide level, which significantly reduces the signals of *C. jejuni* population structure [14]. Other analytical approaches have demonstrated some *C. jejuni* populations have undergone clonal proliferation that exhibit a multi-host profile and may account for a large proportion of clinical strains [15]. A cg/wgMLST genotyping approach demonstrated a lineage of *C. jejuni* (ST2254-9-1) that had low genetic variability compared to other lineages of *C. jejuni* [15]. However, typing schemes available for *C. jejuni* strain classification continue to be challenging. Diversity profiling using a fragment of *porA* gene in *Campylobacter* also identified wide diversity within broiler breeder and broiler flocks, indicating a diverse population of *Campylobacter* has the potential to transmit through the poultry meat production route [16].

Poultry meat has been the predominant source attributable to human campylobacteriosis cases [16, 17]. Further back from the direct exposure to consumers, genetic diversity of *C. jejuni* isolated from chicken carcasses at a slaughter plant included multiple genotypes that are associated with strains found in human infections [18].

Failure to capture the genetic diversity of a *C. jejuni* population within a single human case stool specimen may confound source attribution investigations [9]. Moreover, the replacement of culture-based testing by PCR-based analysis in diagnostic laboratories is eliminating the availability of *C. jejuni* isolates, making epidemiological tracking for outbreak investigation near impossible [19–21].

Currently, the most discriminatory method for investigating strain diversity is by using whole genome sequencing (WGS) and analysing the complete genome [22, 23]. Cao et al. [24] estimated that the *C. jejuni* pangenome consisted of 900 core genes and 4621 accessory genes, based on 173 *C. jejuni* strains, whilst Rossi et al*.* identified 678 core and 2117 accessory genes based on 6526 *C. jejuni* isolates [25]. The different sizes of the pangenomes between these studies can be attributed to the different genomes, software and cut-offs used, but both highlight that *C. jejuni* has a relatively small core genome compared to its large accessory genome. The genetic variation of the genome within the species is thought to be linked to some strains carrying genes associated with increased pathogenicity in human infection [24]. Pathogenicity genes associated with the organism’s ability to survive in adverse conditions and possible host specificity have been reported [24]. The overall diversity of *C. jejuni* therefore requires that a large proportion of the population is analysed in epidemiological investigations [24, 26].

Antimicrobial resistant infections are more difficult to treat, can last longer, and can cause further complications. This increases the costs of healthcare expenses and may further disseminate resistant *Campylobacter* in the community [25, 27]. The WHO has categorised fluoroquinolone resistant *Campylobacter* as a priority list pathogen and classified it as a public health threat [28]. Moreover, in recent years, *C. jejuni* derived from human and chicken specimens have been found to contain resistance to β-lactam and tetracycline antibiotics, which are widely used in human medicine [29, 30]. Since *Campylobacter* is known to exchange genetic material [36], including antimicrobial resistance genes (ARG), the inclusion of resistance determinants is another indicator of intraspecies diversity.

Genetic diversity among multiple isolates can also be described by mapping DNA sequences to a reference genome of the same species to identify variable sites that display single nucleotide polymorphisms (SNPs) [31]. However, SNP analysis for *C. jejuni* has drawbacks as this strategy treats horizontal genetic exchange, locally grouped SNPs acquired in a single event, in the same way as dispersed repeats acquired by multiple events [32]. Horizontal gene exchange is common between *C. jejuni* strains [33] and so standard SNP analysis without removing putative recombinations is likely to overestimate genetic distance between isolates. *Campylobacter* have high frameshift rates that can contribute to genetic diversity and host adaptation through phase variable gene expression [34].

The aims of this study were to investigate the intraspecies genetic variation of a *C. jejuni* population at the pangenome level within patients that presented with gastroenteritis and evaluate whether or not this diversity could have been accumulated since the estimated onset of campylobacteriosis.

## Results

### Patient and *Campylobacter* characterisation

One diarrhoeal stool specimen from four PCR-verified campylobacteriosis patients were cultured for *Campylobacter* using direct-plating and stool filtration prior to plating. Each patient presented with gastroenteritis with three patients presented acute diarrhoea while one patient (patient 3) presented with prolonged diarrhoea for 2 weeks prior to specimen submission. One patient reported travel prior to infection onset (patient 4). Patient age ranged from 7 to 80 years (Additional file 4: Table S1).

A total of 92 *C. jejuni* isolates were recovered (Table 1) with 40% (37/92) of isolates originating from direct plating and 60% (56/92) of isolates originating from stool filtrates. The number of isolates per patient specimen ranged from 13 to 30 isolates; all 92 were classified as *C. jejuni*. A quality check of the 92 *C. jejuni* assembled genomes revealed no evidence of mixed specimens*.* For one patient (patient 3), *C. jejuni* was only isolated using the filtration method, whilst for the other three patients, *C. jejuni* was isolated using a combination of direct and filtration methods. For the three stool specimens where *C. jejuni* were cultured using both methods, no genes, SNPs or frameshifts were associated with method of detection.

**Table 1:** *Campylobacter* recovery, sequence types and antimicrobial resistance in stool specimens of four patients

Patient identifier (total number isolates cultured)	Culture method (*n*)	*Campylobacter* species (*n*)	MLST (*n*)	Acquired resistance gene		Chromosomal point mutation	
				Resistance gene profile (*n*)	Predicted resistance phenotype (*n*)	*gyrA* mutation (*n*)	Predicted resistance phenotype (*n*)
Patient 1 (n = 30)	Direct (15)	*C. jejuni* (15)	ST-61 (15)	*bla*_OXA-61_ (15)	Ampicillin (15)	–	–
	Filtered (15)	*C. jejuni* (15)	ST-61 (15)	*bla*_OXA-61_ (15)	Ampicillin (15)	–	–
Patient 2 (n = 23)	Direct (9)	*C. jejuni* (9)	ST-2066 (9)	*tet*(O) (9)	Tetracycline (9)	T86I (9)	Quinolone (9)
	Filtered (14)	*C. jejuni* (14)	ST-2066 (14)	*tet*(O) (14)	Tetracycline (14)	T86I (14)	Quinolone (14)
Patient 3 (n = 13)	Direct (0)	–	–	–	–	–	–
	Filtered (13)	*C. jejuni* (12), *C. jejuni* (1)	ST-51 (12), ST-354 (1)	*bla*_OXA-61_*, tet*(O) (12), *bla*_OXA-61_*, tet*(O) (1)	Ampicillin, Tetracycline (12), Ampicillin, Tetracycline (11)	T86I (1)	Quinolone (1)
Patient 4 (n = 26)	Direct (13)	*C. jejuni* (13)	ST-21 (13)	*bla*_OXA-61_ (13)	Ampicillin (13)	T86I (13)	Quinolone (13)
	Filtered (13)	*C. jejuni* (13)	ST-21 (13)	*bla*_OXA-61_ (13)	Ampicillin (13)	T86I (13)	Quinolone (13)

*MLST* Multilocus sequence type, *ST* sequence type, “*- *“ No isolates recovered, (*n)* number of isolates

For three of the four patients a single *C. jejuni* sequence type (ST) -61 (patient 1), ST-2066 (patient 2) and ST-21 (patient 4) was detected within each patient specimen while for patient 3, two different STs [ST-51 (n = 12) and ST-354 (n = 1)] were identified (Table 1).

### SNP and frameshift analysis

**Fig. 1:**
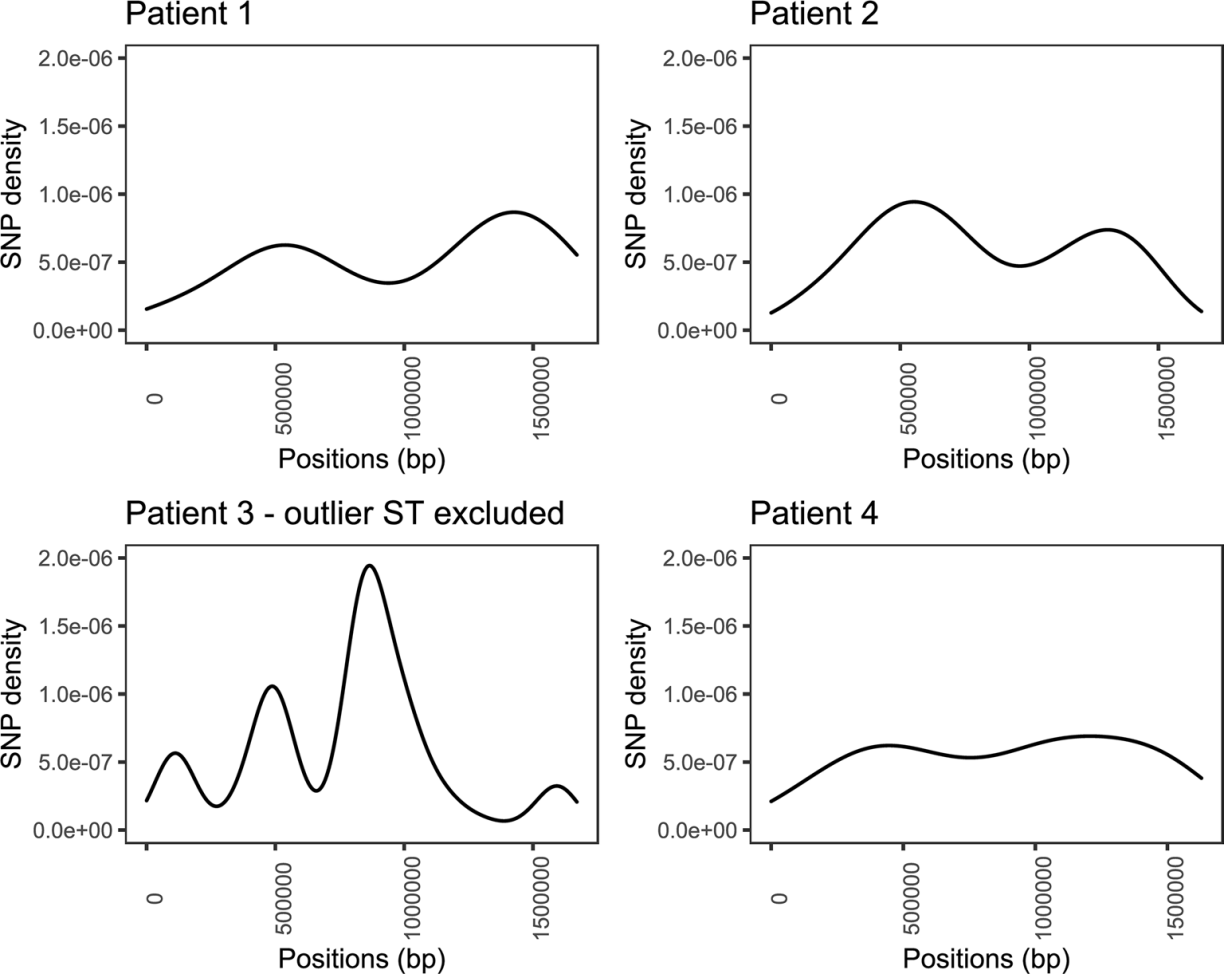
Density plots of single nucleotide polymorphisms number in four patients’ *C. jejuni* along the length of the reference genomes (ST-354 excluded)

**Fig. 2:**
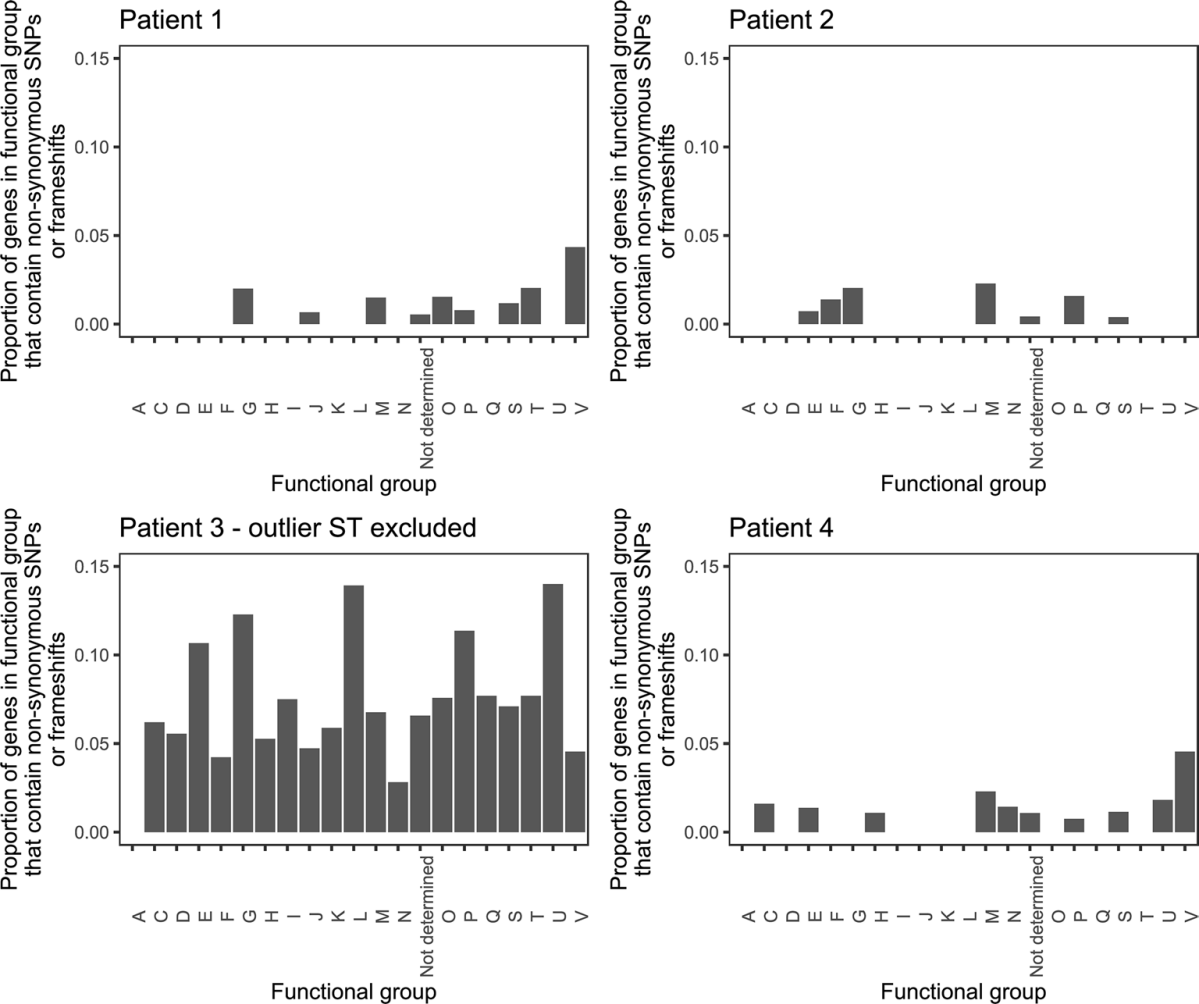
Proportion of gene functional groups (Additional file 4: Table S1) that contained a non-synonymous SNP or frameshift in 91 *C. jejuni* isolates from four patients (ST-354 excluded)

**Fig. 3:**
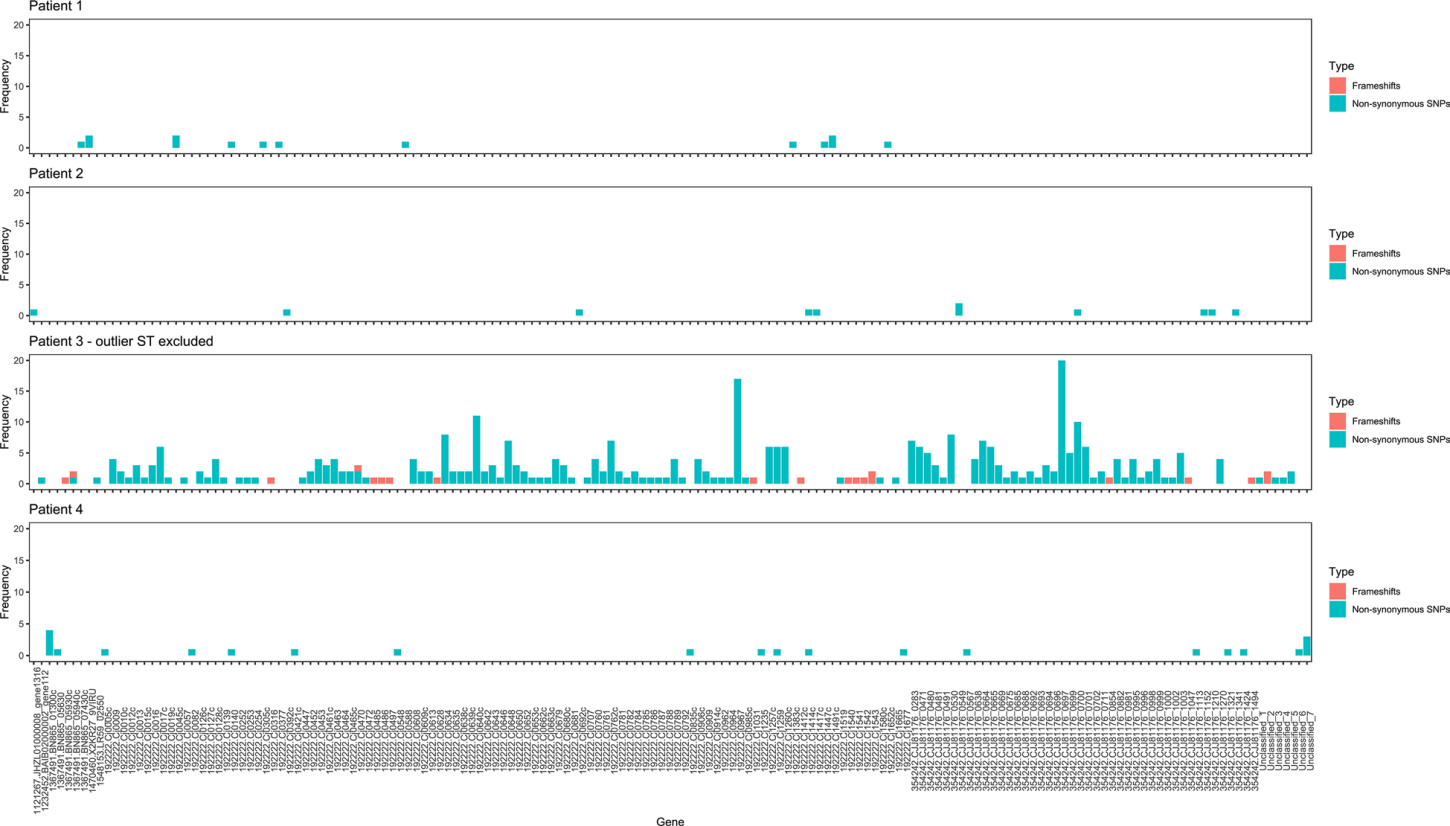
Number of non-synonymous SNPs and frameshifts in genes belonging to four patients’ *C. jejuni* (ST-354 excluded)

SNP analysis was conducted for 91 genomes, excluding the single ST-354 genome from patient 3. The reference genome used to identify SNPs and frameshifts consisted of CP007191 for patient 1, LR134500 for patient 2, NZ_CP059967 for patient 3 and NZ_CP059969 for patient 4. SNP analysis demonstrated a high density of SNPs in isolates from patient 3, even with the outlier ST-354 excluded, while the density of SNPs for the remaining patients’ isolates remained low (Fig. 1). Gubbins software removed a large number of SNPs from the alignment of genomes from patient 3, indicating a high amount of putative recombination had occurred amongst the ST-51 isolates from this patient. Isolates collected from patient 3 also contained a large number of frameshifts (Table 2). The proportion of genes containing non-synonymous SNPs or frameshifts was not significantly different between predicted genes with different COG functional groups (Additional file 4: Table S2) in patient 1 (p = 0.621), patient 2 (p = 0.619), patient 3 (p = 0.577) nor patient 4 (p = 0.871) (Fig. 2), however some did contain multiple non-synonymous SNPs and frameshifts within the same gene (Fig. 3). No SNP or frameshift was associated with a method of detection.

**Table 2:** Number of SNPs and frameshifts between *C. jejuni* isolates from stool specimens of four patients

Patient identifier	Number of isolates	All SNPs		Non-recombinant SNPs		Frameshifts		Alignment size (bp)	
		Minimum	Maximum	Minimum	Maximum	Minimum	Maximum	Core	Recombination
Patient 1	30	0	16	0	12	0	0	1207295	184
Patient 2	23	1	38	0	26	0	0	1188869	737
Patient 3 (ST-354 excluded)	12	4	1080	1	43	0	20	1311111	128185
Patient 4	26	0	16	0	12	0	0	1186019	89

ST sequence type, SNPs Single nucleotide polymorphisms

### Pangenome analysis

**Fig. 4:**
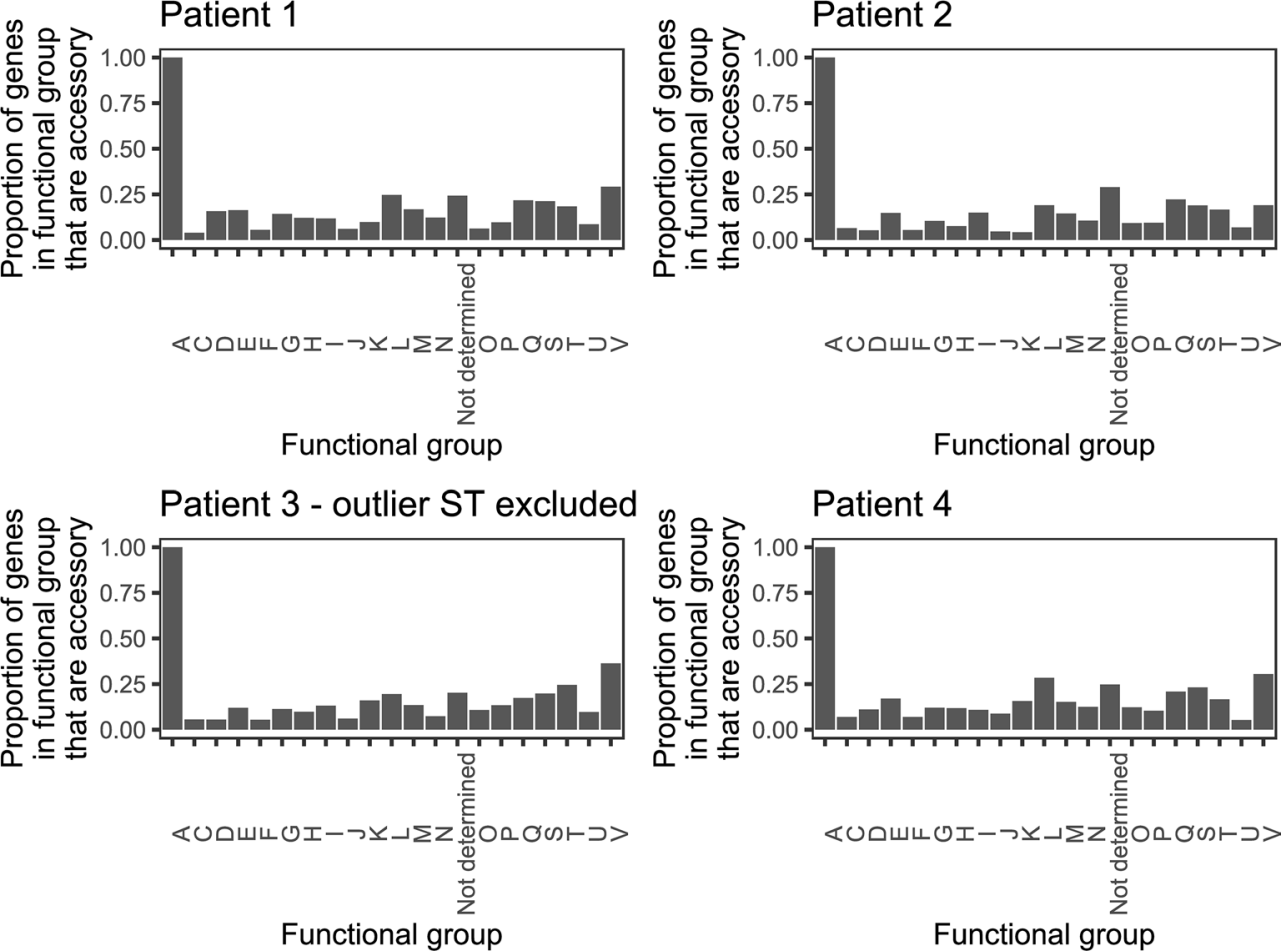
Proportion of gene functional groups (Additional file 4: Table S1) that consisted of accessory genes in four patients’ 91 *C. jejuni* isolates (ST-354 excluded)

For the *C. jejuni* isolates collected from each patient, the pangenomes consisted of a similar proportion of core genes, ranging from 0.84 to 0.86 (Table 3; Additional file 1: Figure S1). Some of the missing genes could be attributed to pseudogenes (Additional file 2: Figure S2). The proportion of genes in the accessory genome significantly differed between functional groups in all patients (p < 1 × 10^–6^) (Fig. 4). The largest difference was between functional group A (RNA processing and modification) and all other functional groups, but for all pangenomes only one gene belonged to this functional group. No gene was associated with method of detection.

**Table 3:** Pangenome structure of 91 *C. jejuni* isolates from stool specimens of four patients

Patient identifier	No. of isolates	Core genome		Accessory genome	
		No. of genes	Proportion of pangenome	No. of genes	Proportion of pangenome
Patient 1	30	1448	0.85	247	0.15
Patient 2	23	1491	0.86	242	0.14
Patient 3	12	1446	0.86	231	0.14
Patient 4	26	1406	0.84	264	0.16
All patients combined	91	1271	0.58	931	0.42

### SNP modelling

The core non-recombinant SNP data amongst isolates from each patient was modelled to determine how many isolates would be required to identify 95% of SNPs in each specimen. The model estimates were close to subsampled estimates (Additional file 3: Figure S3). Modelling found that if we sampled an infinite number of isolates from each specimen we would identify 46–68 core non-recombinant SNPs (Table 4). In addition, to identify 95% of SNPs we would need to specimen 11–81 isolates from each specimen.

**Table 4:** SNP number modelling prediction using known *Campylobacter* genome information in four patients

Patient identifier	Isolates (*N*)	Number of core non-recombinant SNPs (*n*)	Probability of finding a SNP (*p*)	Proportion of SNPs found (*q*)	Number of SNPs if we sampled infinite isolates (*T*)	Number of isolates to identify 95% of SNPs (*N*_*95*_)
1	30	42	0.037	0.672	62	81
2	23	59	0.087	0.877	67	33
3—outlier ST excluded	12	65	0.232	0.958	68	11
4	26	36	0.057	0.780	46	51

### Antimicrobial resistance determinant analysis

**Fig. 5:**
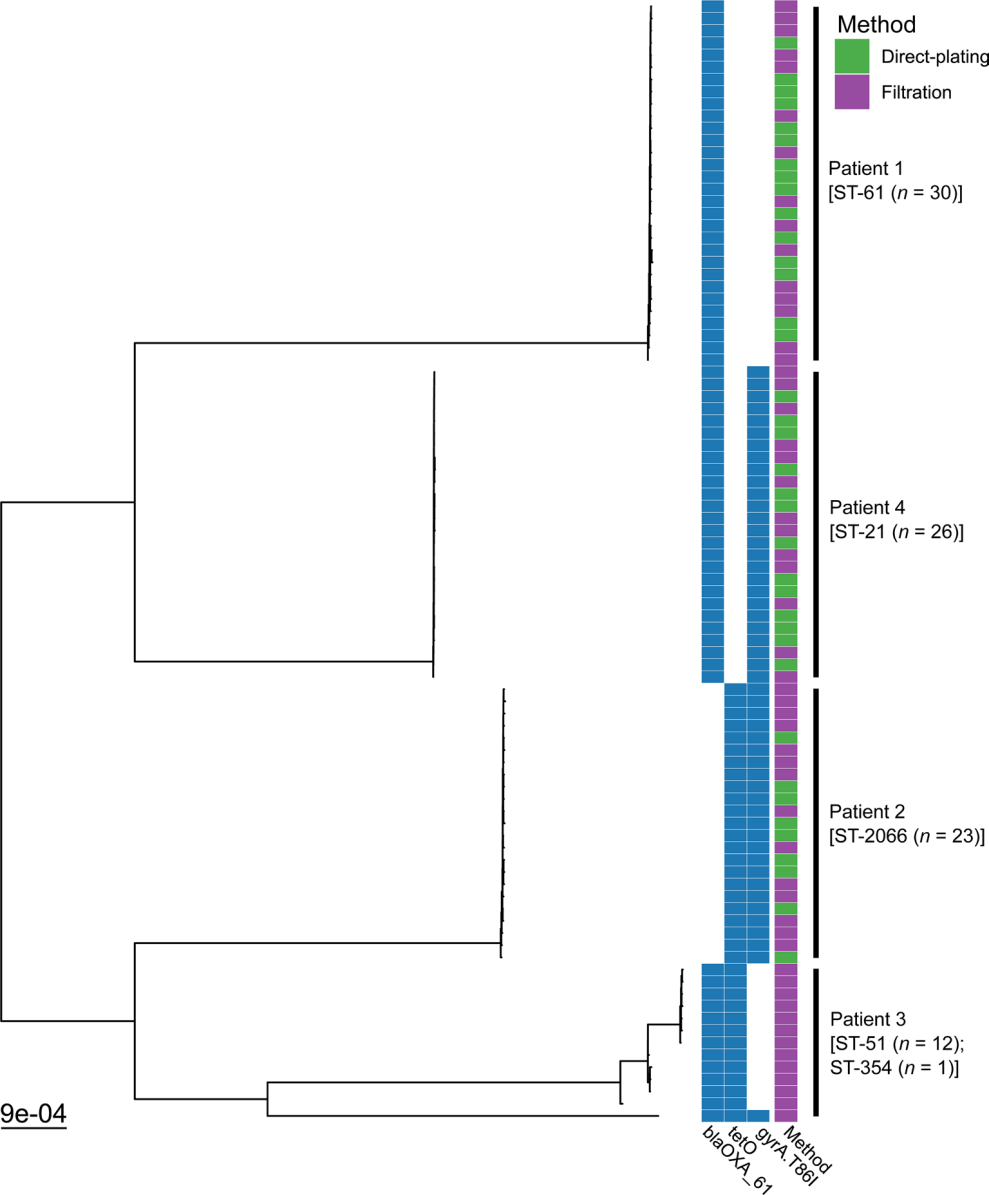
Maximum-likelihood phylogeny based on core gene alignments of 92 *C. jejuni* isolates from four patients, coloured by the presence of AMR determinants and method of detection

In silico antimicrobial resistance determinant analysis found that isolates belonging to the same sequence type contained the same AMR determinants. At least one AMR determinant was found in all STs, with ST-21, ST-51 and ST-2066 containing two AMR determinants and ST-354 containing three AMR determinants. Beta-lactamase conferring resistance gene *bla*_*OXA-61*_ was identified in ST-21, ST-51, ST-61 and ST-354. Tetracycline resistance gene *tet* (O) was identified in ST-51, ST-354 and ST-2066. A single chromosomal mutation of gene *gyrA* T86I, associated with fluoroquinolone resistance, was identified in ST-21, ST-354 and ST-2066. (Table 1; Fig. 5).

## Discussion

In this study we report the genomic diversity of ninety-two *C. jejuni* isolates from four clinical stool specimens at the pangenome level. The *C. jejuni* were cultured using two methods: direct culturing and filtration. For one patient, *C. jejuni* was only isolated using the filtration method, whilst for the other three patients, *C. jejuni* was isolated using a combination of direct and filtration methods. For the three stool specimens where *C. jejuni* were cultured using both methods, no genes or mutations were found to be associated with method of detection. This demonstrates that using the two methodologies increased the chances of culturing *Campylobacter* but did not have an associated effect on the diversity observed.

In this study, we found the maximum number of core non-recombinant SNPs amongst *C. jejuni* isolates belonging to the same sequence type and from the same specimen was 12–43 SNPs. Since *Campylobacter* co-infection is known to occur [9] and genomic diversity generated within a patient through mutation and horizontal gene exchange is frequent [35], outbreak investigations using single colonies are unlikely to capture the genetic diversity of isolates within patients, which could lead to false conclusions [35]. Our modelling of SNPs suggests that to capture 95% of the core non-recombinant SNPs from specimens, up to 80 isolates would need to be collected.

In most cases, human campylobacteriosis is self-limiting, however a significant minority of invasive or chronic infections may require antimicrobial therapy [5, 6]. *Campylobacter* isolates from humans and chickens have evolved resistance to β-lactam and tetracycline antimicrobials [29, 36]. In this study, antimicrobial resistance determinants were associated with β-lactam, tetracycline and quinolone resistance. Previous studies have reported 50–61% of *C. jejuni* isolates with ampicillin resistance [37], 50–100% with tetracycline resistance [38, 39], and 11–40.5% with quinolone resistance [40, 41]. All of *C. jejuni* isolates from patients 1, 3 and 4 contained the *bla*_OXA-61_ gene, responsible for the production of β-lactamase [29] and associated with ampicillin resistance [36]. All *C. jejuni* isolates collected from patients 2 and 4 and the outlier ST-354 in patient 3, contained the chromosomal T86I mutation in *gyrA* associated with quinolone resistance. The single-step T86I amino acid change in the *gyrA* gene found in ST-21, ST-354 and ST-2066 of our study is one of the most prevalent resistance mutations on the chromosome associated with decreased *Campylobacter* susceptibility to fluoroquinolones [42] and so this was an expected finding. There is worldwide concern around quinolone resistance [43–45] threatening the treatment of severe *Campylobacter* infections in humans [46, 47], but transmission routes are not clear—understanding the diversity in a single patient will help us to track the movement of resistance.

*Campylobacter* collected from human patients have been shown to vary in genetic diversity. Dunn et al*.* [48] investigated two *Campylobacter* isolates collected from the same patient from separate stool specimens on subsequent days and identified a single SNP difference between them, whilst Cody et al. [49] investigated twenty patients, comparing two *Campylobacter* isolates collected from separate stool specimens and found three patients with isolates belonging to different sequence types and 17 with isolates belonging to the same sequence type but that differed at 3–14 loci (SNP or frameshift differences). In our study, we isolated two sequence types from one of the patients, and a maximum number of 12–43 core non-recombinant SNPs and 0–20 frameshifts amongst isolates belonging to the same sequence type from the same patient. These results indicate more diverse populations of *Campylobacter* than in the patient described by Dunn et al*.* [48] and some of the patients described by Cody et al. [49]. However, many of the patients described by Cody et al*.* [49] had *Campylobacter* populations with similar diversity measurements as those described in this study, suggesting that only collecting two isolates from a patient is often unable to capture the diversity of *Campylobacter* from patients. Bloomfield et al. [50] and Baker et al. [7] investigated *C. jejuni* collected from the same patients over several years and found a maximum number of 53–84 core non-recombinant SNPs and 18–19 frameshifts amongst the isolates collected from the two patients, and these mutations were associated with genes involved in cell motility and signal transduction. These associations were not observed in this study, suggesting the selection pressures identified by Bloomfield et al. and Baker et al*.* may occur in persistent infections over a longer time period. Frameshifts often occur in genes involved in phase variation and can rapidly accumulate in *C. jejuni* populations. Because of their genetic instability it has been argued that these frameshifts should not be used for public health investigations [22]. However, we also identified core non-recombinant SNPs that are more genetically stable. Bloomfield et al. [50] and Baker et al. [7] both used phylogenetic analysis on core non-recombinant SNPs to determine the date of common ancestor for the *Campylobacter* collected from each patient to estimate when the patients were initially colonised. However, these estimates assume the long-term patients were not colonised with a heterogenous population. Based on the results from this study they may have overestimated the length of time the long-term patients were colonised with *Campylobacter*.

The most distantly related isolates belonging to the same sequence type from each patient shared 12–43 core non-recombinant SNPs. The SNP modelling suggests that we did not detect all SNPs from isolates from the same specimen. Since *C. jejuni* has a substitution rate of 1.5–4.5 × 10^–6^ substitutions site^−1^ year^−1^ [51], that equates to 2–8 SNPs per year, suggesting the isolates may have shared a common ancestor years before the specimens were collected. The exact length of time between when the patient became infected with *C. jejuni* and the collection of stool specimens was unavailable for analysis in this study. In previous studies, patients excreted *Campylobacter* in their stool for up to 2 months post exposure [52]. However, the substitution rate estimates were based on long-term colonisation of human patients, and the substitution rate may be higher for *C. jejuni* during initial infections. Also, those long-term patients were all colonised with ST-45 and it has been proposed that substitution rates may differ significantly between different lineages of *C. jejuni* [53]. Regardless, we believe there is sufficient genetic diversity demonstrated between isolates in this study collected from the same patient to suggest that all patients were colonised with a genetically diverse population of *C. jejuni.* In the case of patient 3, isolates belonging to two sequence types, and in the case of patients 1, 2 and 4, isolates belonging to the same sequence types but genetically diverse in terms of core non-recombinant SNPs. The populations may have become more genetically diverse between infection and specimen collection, but it is unlikely that they accumulated the genetic diversity observed in this study after infection. It is also possible that the patients were exposed to *C. jejuni* on multiple occasions, and since three of the patient specimens contained isolates belonging to the same sequence type, multiple exposures to the same source type may be another exposure scenario. The level of diversity amongst the multiple isolates within a patient described here suggests an infection with a genetically diverse population of *C. jejuni* through  a single source or repeated infections from different sources containing different strains of *C. jejuni,* which have persisted in the human host.

## Conclusions

Using direct plating and filtration culture methods, a total of ninety-two *C. jejuni* isolates were recovered from four different patients with gastroenteritis. For one patient, *C. jejuni* was only isolated using the filtration method, but for the others there were no genetic associations between isolates and method of detection. SNP analysis determined genetic diversity amongst a *C. jejuni* population within a patient’s stool, thereby the *C. jejuni* population may have shared common ancestors before specimens were obtained, indicating that infection could be a result of exposure to a varied population of *C. jejuni*, or a result of subsequent colonisations. The presence of functional genes found in isolates from the same patient varied greatly, as did non-synonymous SNPs and frameshifts in these genes. However, neither the mutation nor the accessory genes were connected to a specific gene functional category which indicates absence of selection at this point of time. The within-patient *C. jejuni* population variance found in this study informs the limitations that exist in studying single isolates per patient specimen and reveals the subtyping genotypic information of campylobacteriosis patients.

## Methods

### Stool specimen collection

Four surplus diarrhoeal stool specimens were collected from the National Health Services Eastern Pathology Alliance (EPA) network diagnostic laboratory, Norwich, UK. Stool specimens represented four separate anonymised patients with gastroenteritis symptoms who submitted specimens to the laboratory in August 2020. Patient specimens were identified in this study as ‘patient 1–4’ and were described further by this patient identifier. Demographic patient information, presentation of diarrhoea and duration of illness prior to specimen collection was recovered retrospectively (Additional file 4: Table S1). *Campylobacter* spp. were initially identified in the stool specimens by the diagnostic laboratory using a rapid automated PCR-based culture-independent testing panel (Gastro Panel 2, EntericBio, Serosep United Kingdom). Once PCR results were confirmed, a 5 mL aliquot of stool was placed into a sterile specimen container and transported to Quadram Institute Bioscience using transport of diagnostic specimens’ guidelines and subjected to culture-based isolation.

### Bacterial isolation and identification

Stool specimens were cultured for *Campylobacter* using modified ISO methods (EN ISO 10272-2019) for detecting and enumerating *Campylobacter* [54] by direct plating and by filtration of stool prior to plating. For the direct plating, a 10 μl aliquot of each stool specimen was directly plated onto modified charcoal-cefoperazone deoxycholate agar (mCCDA) with cefoperazone and amphotericin B supplements (Oxoid, Hampshire, United Kingdom). In parallel, a 4 ml aliquot of each stool specimen was emulsified in 4 ml of phosphate-buffered saline (PBS) and filtered through a 0.65 μm syringe filter (Sartorius, Göttingen, Germany), before 10 µl was inoculated onto a mCCDA plate. All plates throughout the protocol were incubated in a microaerophilic atmosphere using anaerobic jars with CampyGen 2.5 L sachet (Oxoid, Hampshire, United Kingdom) at 37 °C for 48 h. *C. jejuni* strain 81116 was used as a positive control throughout the protocol.

Once incubated, up to 30 suspected *Campylobacter* colonies per patient specimen were sub-cultured onto mCCDA for purification, and further sub-cultured onto Columbia agar with 5% horse blood (Oxoid, Hampshire, United Kingdom). Colony morphology, microscopy, and oxidase test (Thermo Fisher Scientific, Loughborough, United Kingdom) were utilised to confirm presumptive *Campylobacter* isolates.

### Genome extraction and library preparation

DNA from each isolate was extracted using Maxwell RSC Cultured Cells DNA Kits (Promega, Madison, Wisconsin, USA) according to the manufacturer instructions. A modified library preparation was utilised for high throughput sequencing. Libraries for sequencing were prepared using the Illumina DNA Prep (Illumina Ltd, Cambridge, United Kingdom) as previously described [55]. Sequencing was performed on the Illumina Nextseq500 platform using a mid-output flowcell (NSQ^®^ 500 Mid Output KT v2 (300 CYS) Illumina, Cambridge, United Kingdom), producing 2 × 150 bp paired-end reads.

### Genome analysis

Illumina raw reads were trimmed using Trimmomatic v0.38 [56] and assembled using Spades v3.12.0 [57]. Centrifuge v1.0.4 [58] analysis was performed on the trimmed reads to predict bacterial genus and species. The quality of the assemblies was analysed using Quast v5.0.0 [59], CheckM v1.1.3 [60] and aligning reads to the assembly using the Burrows-Wheeler aligner (BWA) v0.7.17 [61].

MLST was conducted using ARIBA v2.14.4 [62] with the *C. jejuni* pubMLST database to predict the sequence type (ST) [63]. Antimicrobial resistance (AMR) genes were identified with ARIBA and the ResFinder [64] database. A custom database consisting of the 23S, *gyrA* and *gyrB* genes from the NC_002163 *C. jejuni* reference genome was used to identify known mutations conferring macrolide and fluoroquinolone resistance respectively. This database was uploaded to GitHub (https://github.com/samuelbloomfield/C_jejuni_point_mutation_database). StarAMR v0.8.0 [65] was used to confirm the AMR determinants identified. StarAMR identified *bla*_OXA-61_ in all isolates from patients 1, 3 and 4, but ARIBA called the gene interrupted in some of these isolates. We compared the gene sequences of the intact and interrupted *bla*_OXA-61_ genes according to ARIBA and found them to be identical, so used StarAMR results for the *bla*_OXA-61_ genes. For the other AMR determinants, StarAMR and ARIBA gave concordant results.

ReferenceSeeker v1.8.0 [66] was used to identify the best reference for isolates from each specimen. Single Nucleotide Polymorphism (SNP) analysis was completed using Snippy v4.3.2 (https://github.com/tseemann/snippy) to align reads from each *C. jejuni* genome to the *C. jejuni* reference genomes. Gubbins v2.3.1 [32] was used to remove areas of putative recombination from full alignments. RaxML v2.3.1 [67] was used to form a phylogenetic tree from the full Snippy alignment using a Generalized Time Reversible model [68].

### Phase variation analysis

Tatajuba v1.0.2 [69] was used to align the trimmed reads of all the isolates to the reference genomes and identify tracts that differed in size between the isolates. A cut-off of 0.90 was used when comparing frameshifts between *C. jejuni* isolates to account for potential small proportions of sequencing errors. eggNOG v5 [70] was used to predict the function of the genes in the reference genome. Fisher’s Exact test, computed using 10^6^ Monte Carlo Markov Chains iterations, was used to determine differences between the proportion of each gene in each functional group (Additional file 4: Table S2) that contained a non-synonymous SNP or frameshift, with p-value < 0.05 regarded as statistically significant. All statistical analyses were performed using R v3.6 [71].

### Pangenome analysis

Assemblies were annotated using Prokka v1.14.6 [72]. The coding-DNA sequences (CDSs) of these assemblies were extracted and a database was formed after removing duplicates using CD-HIT v4.8.1 (https://github.com/weizhongli/cdhit). ARIBA was used to search for the presence of these CDSs. The report files were concatenated, and pseudogenes were identified as previously described by Mather et al*.* [73]. CDSs that were found in more than 95% of *C. jejuni* isolates from a patient specimen were regarded as part of the core genome, whilst the rest were regarded as part of the accessory genome. eggNOG was used to estimate the function of the pan genome and Fisher’s Exact test, computed using 10^6^ Monte Carlo Markov Chains iterations, was used to determine differences between the proportion of accessory genes in each functional group, with p-value < 0.05 regarded as statistically significant. CDSs, SNPs and frameshifts associated with method of detection (direct plating vs filtration) were investigated by searching each patient’s *Campylobacter* pangenome for CDSs or these mutations found in > 95% of isolates identified through one method and < 5% of isolates identified through the other method.

### SNP modelling

The number of isolates required to identify a proportion of SNPs shared amongst isolates from a specimen was calculated by determining the probability of finding a single SNP (*p*). This was calculated by taking a SNP (*i*), counting the number of isolates that contain this SNP (*x*) and dividing by the number of isolates investigated (*N*). The process was repeated for all SNPs shared amongst the isolates (*n*). The arithmetic mean of these proportions was then calculated (Eq. 1). This process assumes every SNP is equally likely to be found, so we only applied this to core non-recombinant SNPs.

1 $$p\, = \,\frac{1}{n}\sum\limits_{i = 1}^{n} {\frac{{x_{i} }}{N}}$$

The proportion of SNPs found (*q*) was then calculated from the probability of finding a single SNP (*p*) and the number of isolates investigated (*N*) (Eq. 2).

2 $$q\, = \,1 - \left( {1 - p} \right)^{N}$$

The number of SNPs we would likely find if we analysed an infinite number of isolates from a patient *(T*) was then calculated given that we found *n* SNPs from *N* isolates (Eq. 3).

3 $$T\, = \,\frac{n}{{1 - \left( {1 - p} \right)^{N} }}$$

We then rearranged the equation to calculate the number of isolates we would need to specimen (*N*) to identify a number of SNPs (*n*) (Eq. 4). As this is a logarithmic equation, we would need infinite specimens to identify all SNPs, but it does allow us to identify a percentage (e.g. 95%).

4 $$N\, = \,\frac{{\ln \left( {1 - \frac{n}{T}} \right)}}{{\ln \left( {1 - p} \right)}}$$

To test these equations, we applied them to the core non-recombinant SNP datasets from each of the four patients investigated in this study using a range of specimen sizes. We also repeated the SNP analysis for each dataset using a range of specimen sizes. For each specimen size, 100 combinations of isolates were randomly selected with replacement, unless less than 100 combinations were possible, where all combinations were used. For each combination, the number of core non-recombinant SNPs was calculated. The mean and 95% confidence intervals were calculated for each specimen size and compared to the model estimates.

## Supplementary Information

**Additional file 1: Figure S1.** Proportion of coding-DNA sequences shared by 91 *C. jejuni* isolates from four patients (ST-354 excluded).

**Additional file 2: Figure S2.** Proportion of accessory genes found in the isolates from the four *C. jejuni* patients (ST-354 excluded) that are due to pseudogenes.

**Additional file 3: Figure S3.** The mean number of SNPs identified amongst isolates collected from the same patient when sampled (blue) with 95% confidence intervals (light-blue) and the model estimates of the number of SNPs identified (red) versus specimen size for the four patients.

**Additional file 4: Table S1. **Patient demographics, travel status and illness presentation. **Table S2. **Function of genes groups found in 92 *C. jejuni *isolates in four patients. **Table S3.** Sequence Read Archive accession numbers and associated metadata of 92 *Campylobacter jejuni* isolates.

## Data Availability

All supporting data, code, and protocols are included in the article or are available as supplementary data files. The online version of this article contains three supplementary figures (Additional file 1: Figure S1, Additional file 2: Figure S2, Additional file 3: Figure S3) and three supplementary tables (Additional file 4: Tables S1, S2, S3). All sequenced *C. jejuni* isolate data are available in the National Centre for Biotechnology Information (NCBI) Sequence Read Archive under the Bioproject accession number PRJNA797426 (http://www.ncbi.nlm.nih.gov/bioproject/797426). Sequence read archive (SRA) accession numbers and associated metadata can be found in the supplementary material of this study (Additional file 4: Table S3).

## Abbreviations

AMR: Antimicrobial resistance
*bla*_OXA_: Beta-lactamase oxacillinase gene
CBA: Columbia blood agar
CDSs: Coding-DNA sequences
COG: Clusters of orthologous group
EPA: Eastern pathology laboratory
*gyrA*: DNA gyrase subunit A
HTA: Health technology assessment
ISO: International organization for standardization
mCCDA: Modified charcoal cefoperazone deoxycholate agar
MLST: Multilocus sequence typing
p: Probability of null hypothesis being correct
PBS: Phosphate-buffered saline
PCR: Polymerase chain reaction
SNPs: Single nucleotide polymorphisms
ST: Sequence type
*tet*: Tetracycline gene
UK: United Kingdom
v: Version
WGS: Whole genome sequencing
β-lactam: Beta lactam
μl: Microlitre
μm: Micrometre

## References

[1] Fiedoruk K, Daniluk T, Rozkiewicz D, Oldak E, Prasad S, Swiecicka I (2019). Whole-genome comparative analysis of *Campylobacter jejuni* strains isolated from patients with diarrhea in northeastern Poland. Gut Pathog.

[2] Adak GK, Meakins SM, Yip H, Lopman BA, O'Brien SJ (2005). Disease risks from foods, England and Wales, 1996–2000. Emerg Infect Dis.

[3] Food Standard Agency UK. A microbiological survey of *Campylobacter *contamination in fresh whole UK-produced chilled chickens at retail sale (Y2/3/4). https://www.food.gov.uk/print/pdf/node/680 Accessed 24 June 2022

[4] Food Standard Agency UK. The burden of foodborne disease in the UK 2018. https://www.food.gov.uk/sites/default/files/media/document/the-burden-of-foodborne-disease-in-the-uk_0.pdf. Accessed 24 June 2022

[5] Dahl LG, Joensen KG, Osterlund MT, Kiil K, Nielsen EM (2021). Prediction of antimicrobial resistance in clinical *Campylobacter jejuni* isolates from whole-genome sequencing data. Eur J Clin Microbiol Infect Dis.

[6] Ternhag A, Torner A, Svensson A, Giesecke J, Ekdahl K (2005). Mortality following *Campylobacter* infection: a registry-based linkage study. BMC Infect Dis.

[7] Barker CR, Painset A, Swift C, Jenkins C, Godbole G, Maiden MCJ (2020). Microevolution of *Campylobacter jejuni* during long-term infection in an immunocompromised host. Sci Rep.

[8] European Reference Laboratory. Protocol for identification of *C. jejuni*, *C. coli* and *C. lari* by gel-based PCR. https://www.sva.se/media/ju0l5ios/eurl_protocol-identification-jejuni-coli-lari_gelpcr_v1.pdf. Accessed on 24/06/2022

[9] Richardson JF, Frost JA, Kramer JM, Thwaites RT, Bolton FJ, Wareing DR (2001). Coinfection with *Campylobacter* species: an epidemiological problem?. J Appl Microbiol.

[10] Pebody RG, Ryan MJ, Wall PG (1997). Outbreaks of *Campylobacter* infection: rare events for a common pathogen. Commun Dis Rep CDR Rev.

[11] Truccollo B, Whyte P, Burgess C, Bolton D (2021). Genetic characterisation of a subset of *Campylobacter jejuni* isolates from clinical and poultry sources in Ireland. PLoS ONE.

[12] Berthenet E, Thepault A, Chemaly M, Rivoal K, Ducournau A, Buissonniere A (2019). Source attribution of *Campylobacter jejuni* shows variable importance of chicken and ruminants reservoirs in non-invasive and invasive French clinical isolates. Sci Rep-Uk.

[13] Suerbaum S, Lohrengel M, Sonnevend A, Ruberg F, Kist M (2001). Allelic diversity and recombination in *Campylobacter jejuni*. J Bacteriol.

[14] Sheppard SK, Jolley KA, Maiden MC (2012). A gene-by-gene approach to bacterial population genomics: whole genome MLST of *Campylobacter*. Genes-Basel.

[15] Nennig M, Llarena A-K, Herold M, Mossong J, Penny C, Losch S (2021). Investigating major recurring *Campylobacter jejuni* lineages in Luxembourg using four core or whole genome sequencing typing schemes. Front Cell Infect.

[16] Colles F, Preston S, Barfod KK, Flammer P, Maiden MC, Smith AL (2019). Parallel sequencing of porA reveals a complex pattern of *Campylobacter* genotypes that differs between broiler and broiler breeder chickens. Sci Rep.

[17] Würfel S, Da Silva W, de Oliveira M, Kleinubing N, Lopes G, Gandra E (2019). Genetic diversity of *Campylobacter jejuni* and *Campylobacter coli* isolated from poultry meat products sold on the retail market in Southern Brazil. Poult Sci.

[18] Prendergast DM, Lynch H, Whyte P, Golden O, Murphy D, Gutierrez M (2022). Genomic diversity, virulence and source of *Campylobacter jejuni* contamination in Irish poultry slaughterhouses by whole genome sequencing. J Appl Microbiol.

[19] Langley G, Besser J, Iwamoto M, Lessa FC, Cronquist A, Skoff TH (2015). Effect of culture-independent diagnostic tests on future emerging infections program surveillance. Emerg Infect Dis.

[20] Schierenberg A, Nipshagen MD, Broekhuizen BDL, van de Pol AC, Bruijning-Verhagen PCJ, Kusters JG (2016). Design of the PROUD study: PCR faeces testing in outpatients with diarrhoea. BMC Infect Dis.

[21] Imhoff B, Morse D, Shiferaw B, Hawkins M, Vugia D, Lance-Parker S (2004). Burden of self-reported acute diarrheal illness in foodnet surveillance areas, 1998–1999. Clin Infect Dis.

[22] Llarena AK, Taboada E, Rossi M (2017). Whole-genome sequencing in epidemiology of *Campylobacter jejuni* infections. J Clin Microbiol.

[23] Nadon C, Van Walle I, Gerner-Smidt P, Campos J, Chinen I, Concepcion-Acevedo J (2017). Pulsenet international: vision for the implementation of whole genome sequencing (WGS) for global food-borne disease surveillance. Eurosurveillance.

[24] Cao HC, Xu HX, Ning CH, Xiang L, Ren QF, Zhang TT (2021). Multi-omics approach reveals the potential core vaccine targets for the emerging foodborne pathogen *Campylobacter jejuni*. Front Microbiol.

[25] Broderick N, Walsh KA, O'Brien KK, Smith SS, Harrington P, O'Neill M (2022). Economic burden of antimicrobial resistance: an analysis of the additional bed day costs associated with treating resistant infections in Ireland. Value Health.

[26] Meric G, Yahara K, Mageiros L, Pascoe B, Maiden MCJ, Jolley KA (2014). A reference pan-genome approach to comparative bacterial genomics: identification of novel epidemiological markers in pathogenic *Campylobacter*. Plos ONE.

[27] Dadgostar P (2019). Antimicrobial resistance: implications and costs. Infect Drug Resist.

[28] Tacconelli E, Carrara E, Savoldi A, Harbarth S, Mendelson M, Monnet DL (2018). Discovery, research, and development of new antibiotics: the WHO priority list of antibiotic-resistant bacteria and tuberculosis. Lancet Infect Dis.

[29] Tajada P, Gomez-Graces JL, Alos JI, Balas D, Cogollos R (1996). Antimicrobial susceptibilities of *Campylobacter jejuni* and *Campylobacter coli* to 12 beta-lactam agents and combinations with beta-lactamase inhibitors. Antimicrob Agents Chemother.

[30] Thwaites RT, Frost JA (1999). Drug resistance in *Campylobacter jejuni*, *C. coli*, and *C. lari* isolated from humans in north west England and Wales, 1997. J Clin Pathol.

[31] Bloomfield S, Duong VT, Tuyen HT, Campbell JI, Thomson NR, Parkhill J (2022). Mobility of antimicrobial resistance across serovars and disease presentations in non-typhoidal *Salmonella* from animals and humans in Vietnam. Microb Genom.

[32] Croucher NJ, Finkelstein JA, Pelton SI, Parkhill J, Bentley SD, Lipsitch M (2015). Population genomic datasets describing the post-vaccine evolutionary epidemiology of *Streptococcus pneumoniae*. Sci Data.

[33] Samarth DP, Kwon YM (2020). Horizontal genetic exchange of chromosomally encoded markers between *Campylobacter jejuni* cells. PLoS ONE.

[34] Bayliss CD, Bidmos FA, Anjum A, Manchev VT, Richards RL, Grossier JP (2012). Phase variable genes of *Campylobacter jejuni* exhibit high mutation rates and specific mutational patterns but mutability is not the major determinant of population structure during host colonization. Nucleic Acids Res.

[35] Worby CJ, Lipsitch M, Hanage WP (2014). Within-host bacterial diversity hinders accurate reconstruction of transmission networks from genomic distance data. PLoS Comput Biol.

[36] Thwaites RT, Frost JA (1999). Drug resistance in *Campylobacter jejuni*, C coli, and C lari isolated from humans in north west England and Wales, 1997. J Clin Pathol.

[37] Moore JE, Barton MD, Blair IS, Corcoran D, Dooley JSG, Fanning S (2006). The epidemiology of antibiotic resistance in *Campylobacter*. Microb Infect.

[38] Sahin O, Plummer PJ, Jordan DM, Sulaj K, Pereira S, Robbe-Austerman S (2008). Emergence of a tetracycline-resistant *Campylobacter jejuni* clone associated with outbreaks of ovine abortion in the United States. J Clin Microb.

[39] Gibreel A, Tracz DM, Nonaka L, Ngo TM, Connell SR, Taylor DE (2004). Incidence of antibiotic resistance in *Campylobacter jejuni* isolated in Alberta, Canada, from 1999 to 2002, with special reference to tet (O)-mediated tetracycline resistance. Antimicrob Agents Chemother.

[40] Nachamkin I, Ung H, Li M (2002). Increasing fluoroquinolone resistance in *Campylobacter jejuni*, Pennsylvania, USA 1982–2001. Emerg Infect Dis.

[41] Sierra-Arguello YM, Quedi Furian T, Perdoncini G, Moraes HLS, Salle CTP, Rodrigues LB (2018). Fluoroquinolone resistance in *Campylobacter jejuni* and *Campylobacter coli* from poultry and human samples assessed by PCR-restriction fragment length polymorphism assay. PLoS ONE.

[42] Nachamkin I, Szymanski CM, Blaser MJ (2008). Campylobacter.

[43] Agunos A, Leger D, Avery BP, Parmley EJ, Deckert A, Carson CA (2013). Ciprofloxacin-resistant *Campylobacter* spp in retail chicken, Western Canada. Emerg Infect Dis.

[44] Panzenhagen PHN, Aguiar WS, Frasao BD, Pereira VLD, Abreu DLD, Rodrigues DD (2016). Prevalence and fluoroquinolones resistance of *Campylobacter* and *Salmonella* isolates from poultry carcasses in Rio de Janeiro. Brazil Food Control.

[45] Wysok B, Wojtacka J, Kivisto R (2020). Pathogenicity of *Campylobacter* strains of poultry and human origin from Poland. Int J Food Microbiol.

[46] Espinoza N, Rojas J, Pollett S, Meza R, Patino L, Leiva M (2020). Validation of the T86I mutation in the gyrA gene as a highly reliable real time PCR target to detect fluoroquinolone-resistant *Campylobacter jejuni*. Bmc Infect Dis.

[47] World Health Organization. Critically important antimicrobials for human medicine, 6th revision. Geneva. Switzerland. Availalbe online: https://apps.who.int/iris/bitstream/handle/10665/312266/9789241515528-eng.pdf?ua%3D1 Accessed 05 July 2022.

[48] Dunn SJ, Pascoe B, Turton J, Fleming V, Diggle M, Sheppard SK (2018). Genomic epidemiology of clinical *Campylobacter* spp at a single health trust site. Microb Genom..

[49] Cody AJ, McCarthy ND, van Rensburg MJ, Isinkaye T, Bentley SD, Parkhill J (2013). Real-time genomic epidemiological evaluation of human *Campylobacter* isolates by use of whole-genome multilocus sequence typing. J Clin Microbiol.

[50] Bloomfield SJ, Midwinter AC, Biggs PJ, French NP, Marshall JC, Hayman DTS (2021). Genomic adaptations of *Campylobacter jejuni* to long-term human colonization. Gut Pathogens.

[51] Bloomfield SJ, Midwinter AC, Biggs PJ, French NP, Marshall JC, Hayman DT (2021). Genomic adaptations of *Campylobacter jejuni* to long-term human colonization. Gut Pathog.

[52] Porter IA, Reid TMS (1980). A milk-borne outbreak of *Campylobacter* infection. J Hyg.

[53] Kovanen SM, Kivistö RI, Rossi M, Schott T, Kärkkäinen U-M, Tuuminen T (2014). Multilocus sequence typing (MLST) and whole-genome MLST of *Campylobacter jejuni* isolates from human infections in three districts during a seasonal peak in Finland. J Clin Microb.

[54] Jacobs-Reitsma WF, Jongenburger I, de Boer E, Biesta-Peters EG (2019). Validation by interlaboratory trials of EN ISO 10272-microbiology of the food chain—Horizontal method for detection and enumeration of *Campylobacter* spp—Part 2: colony-count technique. Int J Food Microbiol.

[55] Parker A, Romano S, Ansorge R, Aboelnoer A, Le Gall G, Savva GM (2021). Heterochronic fecal microbiota transfer reverses hallmarks of the aging murine gut, eye and brain. Eye Brain.

[56] Bolger AM, Lohse M, Usadel B (2014). Trimmomatic: a flexible trimmer for Illumina sequence data. Bioinform.

[57] Bankevich A, Nurk S, Antipov D, Gurevich AA, Dvorkin M, Kulikov AS (2012). SPAdes: a new genome assembly algorithm and its applications to single-cell sequencing. J Comput Biol.

[58] Kim D, Song L, Breitwieser FP, Salzberg SL (2016). Centrifuge: rapid and sensitive classification of metagenomic sequences. Genome Res.

[59] Gurevich A, Saveliev V, Vyahhi N, Tesler G (2013). QUAST: quality assessment tool for genome assemblies. Bioinform.

[60] Parks DH, Imelfort M, Skennerton CT, Hugenholtz P, Tyson GW (2015). CheckM: assessing the quality of microbial genomes recovered from isolates, single cells, and metagenomes. Genome Res.

[61] Li H, Durbin R (2009). Fast and accurate short read alignment with burrows-wheeler transform. Bioinform.

[62] Hunt M, Mather AE, Sanchez-Buso L, Page AJ, Parkhill J, Keane JA (2017). ARIBA: rapid antimicrobial resistance genotyping directly from sequencing reads. Microb Genom.

[63] Jolley KA, Bray JE, Maiden MC (2018). Open-access bacterial population genomics: BIGSdb software, the PubMLST org website and their applications. Wellcome Open Res.

[64] Bortolaia V, Kaas RS, Ruppe E, Roberts MC, Schwarz S, Cattoir V (2020). ResFinder 4 0 for predictions of phenotypes from genotypes. J Antimicro Chemother.

[65] Bharat A, Petkau A, Avery BP, Chen JC, Folster JP, Carson CA (2022). Correlation between phenotypic and *in Silico* detection of antimicrobial resistance in *Salmonella enterica* in Canada using Staramr. Microorganisms.

[66] Schwengers O, Hain T, Chakraborty T, Goesmann A (2019). ReferenceSeeker: rapid determination of appropriate reference genomes. BioRxiv.

[67] Stamatakis A (2014). RAxML version 8: a tool for phylogenetic analysis and post-analysis of large phylogenies. Bioinform.

[68] Tavaré S (1986). Some probabilistic and statistical problems in the analysis of DNA sequences. Lect Math Life Sci.

[69] de Oliveira Martins L, Bloomfield S, Stoakes E, Grant AJ, Page AJ, Mather AE (2022). Tatajuba: exploring the distribution of homopolymer tracts. NAR Genom Bioinform.

[70] Huerta-Cepas J, Szklarczyk D, Heller D, Hernandez-Plaza A, Forslund SK, Cook H (2019). eggNOG 5.0: a hierarchical, functionally and phylogenetically annotated orthology resource based on 5090 organisms and 2502 viruses. Nucleic Acids Res.

[71] Team RC. 2013 R: A language and environment for statistical computing.

[72] Seemann T (2014). Prokka: rapid prokaryotic genome annotation. Bioinform.

[73] Mather AE, Phuong TLT, Gao YF, Clare S, Mukhopadhyay S, Goulding DA (2018). New variant of multidrug-resistant *Salmonella enterica* serovar *Typhimurium* associated with invasive disease in immunocompromised patients in Vietnam. Mbio.

