# Supplementary figures and images for: Comparative genomics of Campylobacter jejuni from clinical campylobacteriosis stool specimens
Source: Gut Pathog. 2022 Dec 7;14:45. doi: 10.1186/s13099-022-00520-1 (PMC9727990; doi:10.1186/s13099-022-00520-1)

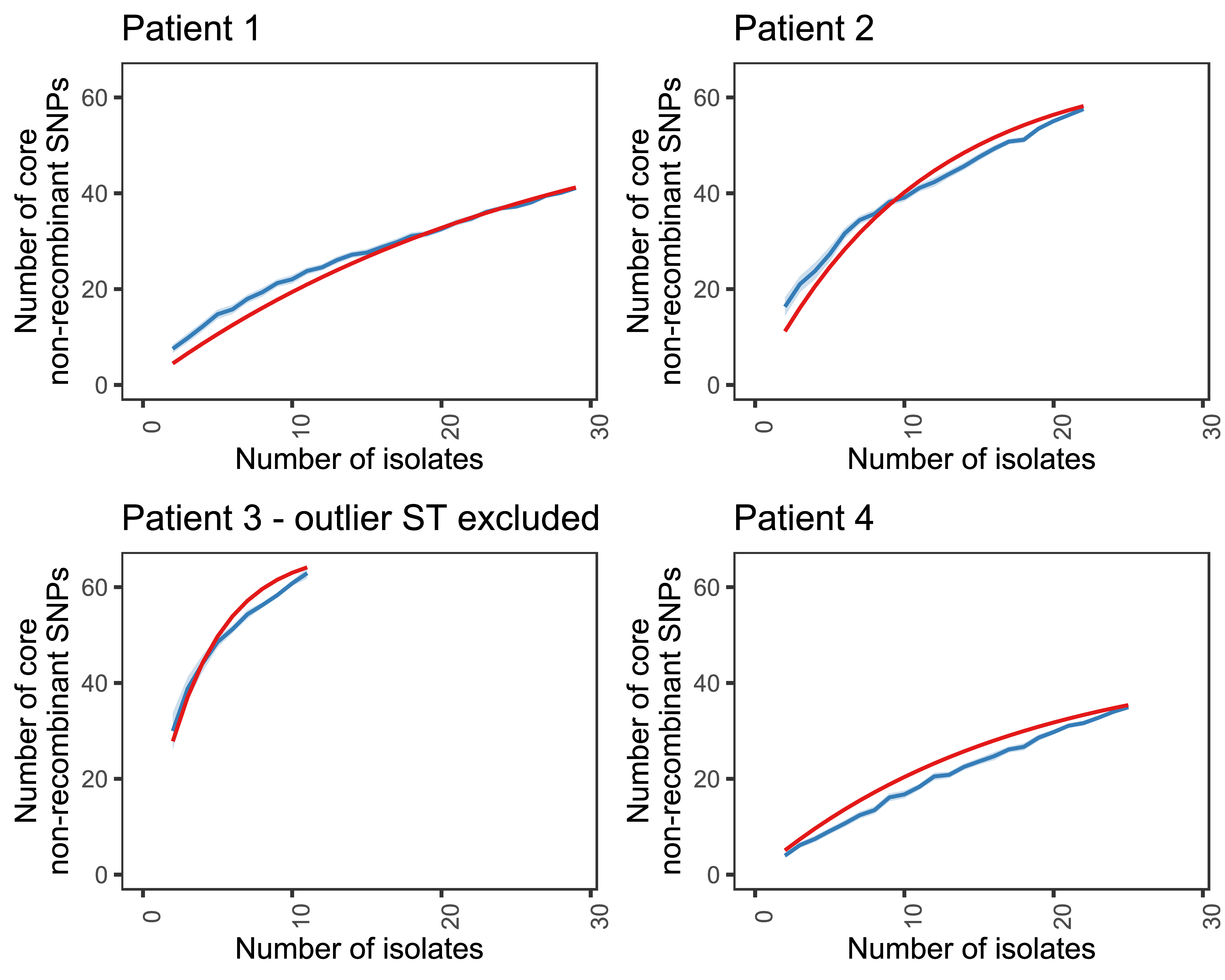

Supplement: Supplementary file 3 — Additional file 3: Figure S3. The mean number of SNPs identified amongst isolates collected from the same patient when sampled (blue) with 95% confidence intervals (light-blue) and the model estimates of the number of SNPs identified (red) versus specimen size for the four patients. [file 13099_2022_520_MOESM3_ESM.tiff]
