# Supplementary material for: Comparative genomics of Campylobacter jejuni from clinical campylobacteriosis stool specimens
Source: Gut Pathog. 2022 Dec 7;14:45. doi: 10.1186/s13099-022-00520-1 (PMC9727990; doi:10.1186/s13099-022-00520-1)
Supplement: Supplementary file 4 — Additional file 4: Table S1. Patient demographics, travel status and illness presentation. Table S2. Function of genes groups found in 92 C. jejuni isolates in four patients. Table S3. Sequence Read Archive accession numbers and associated metadata of 92 Campylobacter jejuni isolates. [file 13099_2022_520_MOESM4_ESM.docx]

**Supplementary material**

**Table S1.** Patient demographics, travel status and illness presentation

| **Patient identifier** | **Demographics (age; gender)** | **Domestic or travel acquired** | **Presentation** | **Symptom duration prior to stool collection** |
| --- | --- | --- | --- | --- |
| Patient 1 | 34; F | Domestic | Acute diarrhoea | Unknown |
| Patient 2 | 7; F | Domestic | Acute diarrhoea | Unknown |
| Patient 3 | 80; F | Domestic | Persistent diarrhoea | 2-weeks |
| Patient 4 | 15; F | Travel | Acute diarrhoea | Unknown |

F – patient identified as female

**Table S2.** Function of genes groups found in 92 *C. jejuni* isolates in four patients.

| **Gene group** | **Functions** |
| --- | --- |
| A | RNA processing and modification |
| B | Chromatin structure and dynamics |
| C | Energy production and conversion |
| D | Cell cycle control, cell division, chromosome partitioning |
| E | Amino acid transport and metabolism |
| F | Nucleotide transport and metabolism |
| G | Carbohydrate transport and metabolism |
| H | Coenzyme transport and metabolism |
| I | Lipid transport and metabolism |
| J | Translation, ribosomal structure and biogenesis |
| K | Transcription |
| L | Replication, recombination and repair |
| M | Cell wall/membrane/envelope biogenesis |
| N | Cell motility |
| O | Post-translational modification, protein turnover, and chaperones |
| P | Inorganic ion transport and metabolism |
| Q | Secondary metabolites biosynthesis, transport, and catabolism |
| R | General function prediction only |
| S | Function unknown |
| T | Signal transduction mechanisms |
| U | Intracellular trafficking, secretion, and vesicular transport |
| V | Defence mechanisms |
| W | Extracellular structures |
| Y | Nuclear structure |
| Z | Cytoskeleton |

**Table S3**. Sequence Read Archive accession numbers and associated metadata of 92 *Campylobacter jejuni* isolates.

| **Sample name** | **SRA number** | **Bioproject accession** | **Organism** | **Host** | **Isolation source** | **Collection date** | **Geographical location** | **Sample type** |
| --- | --- | --- | --- | --- | --- | --- | --- | --- |
| 20EPA011CF1 | SRR18166204 | PRJNA797426 | *Campylobacter jejuni* | Homo sapiens | Stool | 24/08/2020 | United Kingdom: Norwich | Cell Culture |
| 20EPA011CF2 | SRR18166203 | PRJNA797426 | *Campylobacter jejuni* | Homo sapiens | Stool | 24/08/2020 | United Kingdom: Norwich | Cell Culture |
| 20EPA011CF3 | SRR18166202 | PRJNA797426 | *Campylobacter jejuni* | Homo sapiens | Stool | 24/08/2020 | United Kingdom: Norwich | Cell Culture |
| 20EPA011CF4 | SRR18166201 | PRJNA797426 | *Campylobacter jejuni* | Homo sapiens | Stool | 24/08/2020 | United Kingdom: Norwich | Cell Culture |
| 20EPA011CF5 | SRR18166200 | PRJNA797426 | *Campylobacter jejuni* | Homo sapiens | Stool | 24/08/2020 | United Kingdom: Norwich | Cell Culture |
| 20EPA011CF6 | SRR18166199 | PRJNA797426 | *Campylobacter jejuni* | Homo sapiens | Stool | 24/08/2020 | United Kingdom: Norwich | Cell Culture |
| 20EPA011CF7 | SRR18166198 | PRJNA797426 | *Campylobacter jejuni* | Homo sapiens | Stool | 24/08/2020 | United Kingdom: Norwich | Cell Culture |
| 20EPA011CF8 | SRR18166197 | PRJNA797426 | *Campylobacter jejuni* | Homo sapiens | Stool | 24/08/2020 | United Kingdom: Norwich | Cell Culture |
| 20EPA011CF9 | SRR18166196 | PRJNA797426 | *Campylobacter jejuni* | Homo sapiens | Stool | 24/08/2020 | United Kingdom: Norwich | Cell Culture |
| 20EPA011CF10 | SRR18166195 | PRJNA797426 | *Campylobacter jejuni* | Homo sapiens | Stool | 24/08/2020 | United Kingdom: Norwich | Cell Culture |
| 20EPA011CF11 | SRR18166194 | PRJNA797426 | *Campylobacter jejuni* | Homo sapiens | Stool | 24/08/2020 | United Kingdom: Norwich | Cell Culture |
| 20EPA011CF12 | SRR18166193 | PRJNA797426 | *Campylobacter jejuni* | Homo sapiens | Stool | 24/08/2020 | United Kingdom: Norwich | Cell Culture |
| 20EPA011CF13 | SRR18166192 | PRJNA797426 | *Campylobacter jejuni* | Homo sapiens | Stool | 24/08/2020 | United Kingdom: Norwich | Cell Culture |
| 20EPA011CF14 | SRR18166191 | PRJNA797426 | *Campylobacter jejuni* | Homo sapiens | Stool | 24/08/2020 | United Kingdom: Norwich | Cell Culture |
| 20EPA011CF15 | SRR18166190 | PRJNA797426 | *Campylobacter jejuni* | Homo sapiens | Stool | 24/08/2020 | United Kingdom: Norwich | Cell Culture |
| 20EPA011CP1 | SRR18166189 | PRJNA797426 | *Campylobacter jejuni* | Homo sapiens | Stool | 24/08/2020 | United Kingdom: Norwich | Cell Culture |
| 20EPA011CP2 | SRR18166188 | PRJNA797426 | *Campylobacter jejuni* | Homo sapiens | Stool | 24/08/2020 | United Kingdom: Norwich | Cell Culture |
| 20EPA011CP3 | SRR18166187 | PRJNA797426 | *Campylobacter jejuni* | Homo sapiens | Stool | 24/08/2020 | United Kingdom: Norwich | Cell Culture |
| 20EPA011CP4 | SRR18166186 | PRJNA797426 | *Campylobacter jejuni* | Homo sapiens | Stool | 24/08/2020 | United Kingdom: Norwich | Cell Culture |
| 20EPA011CP5 | SRR18166185 | PRJNA797426 | *Campylobacter jejuni* | Homo sapiens | Stool | 24/08/2020 | United Kingdom: Norwich | Cell Culture |
| 20EPA011CP6 | SRR18166184 | PRJNA797426 | *Campylobacter jejuni* | Homo sapiens | Stool | 24/08/2020 | United Kingdom: Norwich | Cell Culture |
| 20EPA011CP7 | SRR18166183 | PRJNA797426 | *Campylobacter jejuni* | Homo sapiens | Stool | 24/08/2020 | United Kingdom: Norwich | Cell Culture |
| 20EPA011CP8 | SRR18166182 | PRJNA797426 | *Campylobacter jejuni* | Homo sapiens | Stool | 24/08/2020 | United Kingdom: Norwich | Cell Culture |
| 20EPA011CP9 | SRR18166181 | PRJNA797426 | *Campylobacter jejuni* | Homo sapiens | Stool | 24/08/2020 | United Kingdom: Norwich | Cell Culture |
| 20EPA011CP10 | SRR18166180 | PRJNA797426 | *Campylobacter jejuni* | Homo sapiens | Stool | 24/08/2020 | United Kingdom: Norwich | Cell Culture |
| 20EPA011CP11 | SRR18166179 | PRJNA797426 | *Campylobacter jejuni* | Homo sapiens | Stool | 24/08/2020 | United Kingdom: Norwich | Cell Culture |
| 20EPA011CP12 | SRR18166178 | PRJNA797426 | *Campylobacter jejuni* | Homo sapiens | Stool | 24/08/2020 | United Kingdom: Norwich | Cell Culture |
| 20EPA011CP13 | SRR18166177 | PRJNA797426 | *Campylobacter jejuni* | Homo sapiens | Stool | 24/08/2020 | United Kingdom: Norwich | Cell Culture |
| 20EPA011CP14 | SRR18166176 | PRJNA797426 | *Campylobacter jejuni* | Homo sapiens | Stool | 24/08/2020 | United Kingdom: Norwich | Cell Culture |
| 20EPA011CP15 | SRR18166175 | PRJNA797426 | *Campylobacter jejuni* | Homo sapiens | Stool | 24/08/2020 | United Kingdom: Norwich | Cell Culture |
| 20EPA012CF1 | SRR18166174 | PRJNA797426 | *Campylobacter jejuni* | Homo sapiens | Stool | 21/08/2020 | United Kingdom: Norwich | Cell Culture |
| 20EPA012CF2 | SRR18166173 | PRJNA797426 | *Campylobacter jejuni* | Homo sapiens | Stool | 21/08/2020 | United Kingdom: Norwich | Cell Culture |
| 20EPA012CF3 | SRR18166145 | PRJNA797426 | *Campylobacter jejuni* | Homo sapiens | Stool | 21/08/2020 | United Kingdom: Norwich | Cell Culture |
| 20EPA012CF4 | SRR18166144 | PRJNA797426 | *Campylobacter jejuni* | Homo sapiens | Stool | 21/08/2020 | United Kingdom: Norwich | Cell Culture |
| 20EPA012CF6 | SRR18166143 | PRJNA797426 | *Campylobacter jejuni* | Homo sapiens | Stool | 21/08/2020 | United Kingdom: Norwich | Cell Culture |
| 20EPA012CF7 | SRR18166142 | PRJNA797426 | *Campylobacter jejuni* | Homo sapiens | Stool | 21/08/2020 | United Kingdom: Norwich | Cell Culture |
| 20EPA012CF8 | SRR18166141 | PRJNA797426 | *Campylobacter jejuni* | Homo sapiens | Stool | 21/08/2020 | United Kingdom: Norwich | Cell Culture |
| 20EPA012CF9 | SRR18166140 | PRJNA797426 | *Campylobacter jejuni* | Homo sapiens | Stool | 21/08/2020 | United Kingdom: Norwich | Cell Culture |
| 20EPA012CF10 | SRR18166139 | PRJNA797426 | *Campylobacter jejuni* | Homo sapiens | Stool | 21/08/2020 | United Kingdom: Norwich | Cell Culture |
| 20EPA012CF11 | SRR18166138 | PRJNA797426 | *Campylobacter jejuni* | Homo sapiens | Stool | 21/08/2020 | United Kingdom: Norwich | Cell Culture |
| 20EPA012CF12 | SRR18166137 | PRJNA797426 | *Campylobacter jejuni* | Homo sapiens | Stool | 21/08/2020 | United Kingdom: Norwich | Cell Culture |
| 20EPA012CF13 | SRR18166136 | PRJNA797426 | *Campylobacter jejuni* | Homo sapiens | Stool | 21/08/2020 | United Kingdom: Norwich | Cell Culture |
| 20EPA012CF14 | SRR18166135 | PRJNA797426 | *Campylobacter jejuni* | Homo sapiens | Stool | 21/08/2020 | United Kingdom: Norwich | Cell Culture |
| 20EPA012CF15 | SRR18166134 | PRJNA797426 | *Campylobacter jejuni* | Homo sapiens | Stool | 21/08/2020 | United Kingdom: Norwich | Cell Culture |
| 20EPA012CP4 | SRR18166133 | PRJNA797426 | *Campylobacter jejuni* | Homo sapiens | Stool | 21/08/2020 | United Kingdom: Norwich | Cell Culture |
| 20EPA012CP5 | SRR18166132 | PRJNA797426 | *Campylobacter jejuni* | Homo sapiens | Stool | 21/08/2020 | United Kingdom: Norwich | Cell Culture |
| 20EPA012CP6 | SRR18166131 | PRJNA797426 | *Campylobacter jejuni* | Homo sapiens | Stool | 21/08/2020 | United Kingdom: Norwich | Cell Culture |
| 20EPA012CP9 | SRR18166130 | PRJNA797426 | *Campylobacter jejuni* | Homo sapiens | Stool | 21/08/2020 | United Kingdom: Norwich | Cell Culture |
| 20EPA012CP10 | SRR18166129 | PRJNA797426 | *Campylobacter jejuni* | Homo sapiens | Stool | 21/08/2020 | United Kingdom: Norwich | Cell Culture |
| 20EPA012CP11 | SRR18166128 | PRJNA797426 | *Campylobacter jejuni* | Homo sapiens | Stool | 21/08/2020 | United Kingdom: Norwich | Cell Culture |
| 20EPA012CP13 | SRR18166127 | PRJNA797426 | *Campylobacter jejuni* | Homo sapiens | Stool | 21/08/2020 | United Kingdom: Norwich | Cell Culture |
| 20EPA012CP14 | SRR18166126 | PRJNA797426 | *Campylobacter jejuni* | Homo sapiens | Stool | 21/08/2020 | United Kingdom: Norwich | Cell Culture |
| 20EPA012CP15 | SRR18166125 | PRJNA797426 | *Campylobacter jejuni* | Homo sapiens | Stool | 21/08/2020 | United Kingdom: Norwich | Cell Culture |
| 20EPA013CF1 | SRR18166124 | PRJNA797426 | *Campylobacter jejuni* | Homo sapiens | Stool | 20/08/2020 | United Kingdom: Norwich | Cell Culture |
| 20EPA013CF2 | SRR18166123 | PRJNA797426 | *Campylobacter jejuni* | Homo sapiens | Stool | 20/08/2020 | United Kingdom: Norwich | Cell Culture |
| 20EPA013CF3 | SRR18166122 | PRJNA797426 | *Campylobacter jejuni* | Homo sapiens | Stool | 20/08/2020 | United Kingdom: Norwich | Cell Culture |
| 20EPA013CF4 | SRR18166121 | PRJNA797426 | *Campylobacter jejuni* | Homo sapiens | Stool | 20/08/2020 | United Kingdom: Norwich | Cell Culture |
| 20EPA013CF5 | SRR18166120 | PRJNA797426 | *Campylobacter jejuni* | Homo sapiens | Stool | 20/08/2020 | United Kingdom: Norwich | Cell Culture |
| 20EPA013CF6 | SRR18166119 | PRJNA797426 | *Campylobacter jejuni* | Homo sapiens | Stool | 20/08/2020 | United Kingdom: Norwich | Cell Culture |
| 20EPA013CF7 | SRR18166118 | PRJNA797426 | *Campylobacter jejuni* | Homo sapiens | Stool | 20/08/2020 | United Kingdom: Norwich | Cell Culture |
| 20EPA013CF8 | SRR18166117 | PRJNA797426 | *Campylobacter jejuni* | Homo sapiens | Stool | 20/08/2020 | United Kingdom: Norwich | Cell Culture |
| 20EPA013CF9 | SRR18166116 | PRJNA797426 | *Campylobacter jejuni* | Homo sapiens | Stool | 20/08/2020 | United Kingdom: Norwich | Cell Culture |
| 20EPA013CF10 | SRR18166115 | PRJNA797426 | *Campylobacter jejuni* | Homo sapiens | Stool | 20/08/2020 | United Kingdom: Norwich | Cell Culture |
| 20EPA013CF11 | SRR18166114 | PRJNA797426 | *Campylobacter jejuni* | Homo sapiens | Stool | 20/08/2020 | United Kingdom: Norwich | Cell Culture |
| 20EPA013CF13 | SRR18166172 | PRJNA797426 | *Campylobacter jejuni* | Homo sapiens | Stool | 20/08/2020 | United Kingdom: Norwich | Cell Culture |
| 20EPA013CF14 | SRR18166171 | PRJNA797426 | *Campylobacter jejuni* | Homo sapiens | Stool | 20/08/2020 | United Kingdom: Norwich | Cell Culture |
| 20EPA015CF2 | SRR18166170 | PRJNA797426 | *Campylobacter jejuni* | Homo sapiens | Stool | 21/08/2020 | United Kingdom: Norwich | Cell Culture |
| 20EPA015CF3 | SRR18166169 | PRJNA797426 | *Campylobacter jejuni* | Homo sapiens | Stool | 21/08/2020 | United Kingdom: Norwich | Cell Culture |
| 20EPA015CF4 | SRR18166168 | PRJNA797426 | *Campylobacter jejuni* | Homo sapiens | Stool | 21/08/2020 | United Kingdom: Norwich | Cell Culture |
| 20EPA015CF5 | SRR18166167 | PRJNA797426 | *Campylobacter jejuni* | Homo sapiens | Stool | 21/08/2020 | United Kingdom: Norwich | Cell Culture |
| 20EPA015CF6 | SRR18166166 | PRJNA797426 | *Campylobacter jejuni* | Homo sapiens | Stool | 21/08/2020 | United Kingdom: Norwich | Cell Culture |
| 20EPA015CF7 | SRR18166165 | PRJNA797426 | *Campylobacter jejuni* | Homo sapiens | Stool | 21/08/2020 | United Kingdom: Norwich | Cell Culture |
| 20EPA015CF8 | SRR18166164 | PRJNA797426 | *Campylobacter jejuni* | Homo sapiens | Stool | 21/08/2020 | United Kingdom: Norwich | Cell Culture |
| 20EPA015CF9 | SRR18166163 | PRJNA797426 | *Campylobacter jejuni* | Homo sapiens | Stool | 21/08/2020 | United Kingdom: Norwich | Cell Culture |
| 20EPA015CF10 | SRR18166162 | PRJNA797426 | *Campylobacter jejuni* | Homo sapiens | Stool | 21/08/2020 | United Kingdom: Norwich | Cell Culture |
| 20EPA015CF11 | SRR18166161 | PRJNA797426 | *Campylobacter jejuni* | Homo sapiens | Stool | 21/08/2020 | United Kingdom: Norwich | Cell Culture |
| 20EPA015CF12 | SRR18166160 | PRJNA797426 | *Campylobacter jejuni* | Homo sapiens | Stool | 21/08/2020 | United Kingdom: Norwich | Cell Culture |
| 20EPA015CF13 | SRR18166159 | PRJNA797426 | *Campylobacter jejuni* | Homo sapiens | Stool | 21/08/2020 | United Kingdom: Norwich | Cell Culture |
| 20EPA015CF14 | SRR18166158 | PRJNA797426 | *Campylobacter jejuni* | Homo sapiens | Stool | 21/08/2020 | United Kingdom: Norwich | Cell Culture |
| 20EPA015CP1 | SRR18166157 | PRJNA797426 | *Campylobacter jejuni* | Homo sapiens | Stool | 21/08/2020 | United Kingdom: Norwich | Cell Culture |
| 20EPA015CP2 | SRR18166156 | PRJNA797426 | *Campylobacter jejuni* | Homo sapiens | Stool | 21/08/2020 | United Kingdom: Norwich | Cell Culture |
| 20EPA015CP4 | SRR18166155 | PRJNA797426 | *Campylobacter jejuni* | Homo sapiens | Stool | 21/08/2020 | United Kingdom: Norwich | Cell Culture |
| 20EPA015CP5 | SRR18166154 | PRJNA797426 | *Campylobacter jejuni* | Homo sapiens | Stool | 21/08/2020 | United Kingdom: Norwich | Cell Culture |
| 20EPA015CP6 | SRR18166153 | PRJNA797426 | *Campylobacter jejuni* | Homo sapiens | Stool | 21/08/2020 | United Kingdom: Norwich | Cell Culture |
| 20EPA015CP7 | SRR18166152 | PRJNA797426 | *Campylobacter jejuni* | Homo sapiens | Stool | 21/08/2020 | United Kingdom: Norwich | Cell Culture |
| 20EPA015CP9 | SRR18166151 | PRJNA797426 | *Campylobacter jejuni* | Homo sapiens | Stool | 21/08/2020 | United Kingdom: Norwich | Cell Culture |
| 20EPA015CP10 | SRR18166150 | PRJNA797426 | *Campylobacter jejuni* | Homo sapiens | Stool | 21/08/2020 | United Kingdom: Norwich | Cell Culture |
| 20EPA015CP11 | SRR18166149 | PRJNA797426 | *Campylobacter jejuni* | Homo sapiens | Stool | 21/08/2020 | United Kingdom: Norwich | Cell Culture |
| 20EPA015CP12 | SRR18166148 | PRJNA797426 | *Campylobacter jejuni* | Homo sapiens | Stool | 21/08/2020 | United Kingdom: Norwich | Cell Culture |
| 20EPA015CP13 | SRR18166147 | PRJNA797426 | *Campylobacter jejuni* | Homo sapiens | Stool | 21/08/2020 | United Kingdom: Norwich | Cell Culture |
| 20EPA015CP14 | SRR18166146 | PRJNA797426 | *Campylobacter jejuni* | Homo sapiens | Stool | 21/08/2020 | United Kingdom: Norwich | Cell Culture |
| 20EPA015CP15 | SRR18166204 | PRJNA797426 | *Campylobacter jejuni* | Homo sapiens | Stool | 21/08/2020 | United Kingdom: Norwich | Cell Culture |
